# Bevacizumab Attenuates Hepatic Fibrosis in Rats by Inhibiting Activation of Hepatic Stellate Cells

**DOI:** 10.1371/journal.pone.0073492

**Published:** 2013-08-30

**Authors:** Yangqing Huang, Helin Feng, Tong Kan, Bin Huang, Minfeng Zhang, Yesheng Li, Changying Shi, Mengchao Wu, Yunquan Luo, Jiamei Yang, Feng Xu

**Affiliations:** 1 Department of Special Treatment, Eastern Hepatobiliary Surgery Hospital, Second Military Medical University, Shanghai, China; 2 Department of Hepatobiliary Surgery, Shuguang Hospital, Shanghai, China; 3 The Second Department of Biliary Surgery, Eastern Hepatobiliary Surgery Hospital, Second Military Medical University, Shanghai, China; 4 Department of Radiology, Eastern Hepatobiliary Surgery Hospital, Second Military Medical University, Shanghai, China; 5 Department of Hepatic Surgery, Eastern Hepatobiliary Surgery Hospital, Second Military Medical University, Shanghai, China; 6 Department of Orthopedics, The Fourth Affiliated Hospital of Hebei Medical University, Shijiazhuang, Hebei, China; CIMA. University of Navarra, Spain

## Abstract

Angiogenesis is a fundamental part of the response to tissue injury, which is involved in the development of hepatic fibrosis. Vascular endothelial growth factor plays an important role in angiogenesis. The expression of VEGF is increased during hepatic fibrogenesis and correlates with the micro-vessel density. In this study, we investigated the effects of bevacizumab, an anti-angiogenetic drug, on the formation of hepatic fibrosis. We found that bevacizumab could attenuate the development of hepatic fibrosis and contribute to the protection of liver function. Bevacizumab was also found to downregulate the expression α-SMA and TGF-β1, which have been reported to be profibrogenic genes *in vivo*. We also observed that the expression of VEGF increased significantly during the development of hepatic fibrosis and CCl_4_ was found to induce hepatocytes to secrete VEGF, which led to the activation and proliferation of HSCs. Bevacizumab was also found to block the effects of the hepatocytes on the activation and proliferation of HSCs. Our results suggest that bevacizumab might alleviate liver fibrosis by blocking the effect of VEGF on HSCs. Bevacizumab might be suitable as a potential agent for hepatic fibrosis therapy.

## Introduction

Hepatic fibrosis is characterized by excess production and deposition of extracellular matrix (ECM), which leads to loss of liver function and structure disruption of liver tissue[1], [2]. Angiogenesis is a complex process leading to generation of new blood vessels from pre-existing blood vessels[3], [4]. Angiogenesis is known to play a critical role in pathological settings like chronic inflammatory and tumor growth[5], [6], [7]. Vascular endothelial growth factor (VEGF) is considered to be the central angiogenic factor during chronic liver injury. The present study demonstrates that expression of VEGF-A is up-regulated during liver fibrosis, and its expression is increased in activated hepatic stellate cells (HSCs) [5], [6], [8], [9].

Hepatic stellate cells (HSCs) play an important role in the development of hepatic fibrosis. HSCs are considered as a key target in anti-fibrotic therapy because of their role in ECM accumulation[10], [11], [12], [13]. Evidence indicates that activated HSCs can express VEGF and VEGF receptors in the liver after carbon tetrachloride (CCl_4_) intoxication[14], [15]. Inflammatory mediators cause the HSCs to differentiate into myofibroblasts. They play a role in angiogenesis and act by releasing the proangiogenic mediators VEGF and angiopoietin-1 during the development of liver fibrosis[16], [17].

Bevacizumab, a full-length humanized monoclonal antibody, is a therapeutic candidate suitable for use as a direct inhibitor of angiogenesis. Its antiangiogenic efficacy is attributable to its ability to bind and neutralize all isoforms of VEGF-A [18]. Bevacizumab has been used to treat metastatic colorectal and metastatic breast cancer[19], [20]. Recent studies suggest that anti-angiogenic therapies can prevent liver fibrosis[5], [21], [22], [23], [24], [25]. Bevacizumab has a potent anti-fibrotic effect in human Tenon's fibrosis by inhibiting VEGF-A. However, to date, the effects of bevacizumab in liver fibrosis are largely unknown. These observations have led us to hypothesize that bevacizumab may inhibit the development of pathological angiogenesis in fibrotic tissue and influence the progress of hepatic fibrosis.

In this study, we investigated the effects of bevacizumab on liver fibrosis. Carbon tetrachloride was used to establish a hepatic fibrosis animal model suitable for observation of the effect of bevacizumab *in vivo*. We then, examined the role of bevacizumab in the proliferation and activation of HSCs *in vitro*. Our results demonstrated that bevacizumab administration could alleviate liver fibrosis by inhibiting activation and proliferation of HSCs.

## Results

### Effects of bevacizumab on hepatic fibrosis induced by CCl_4_ in rats

**Figure 1:**
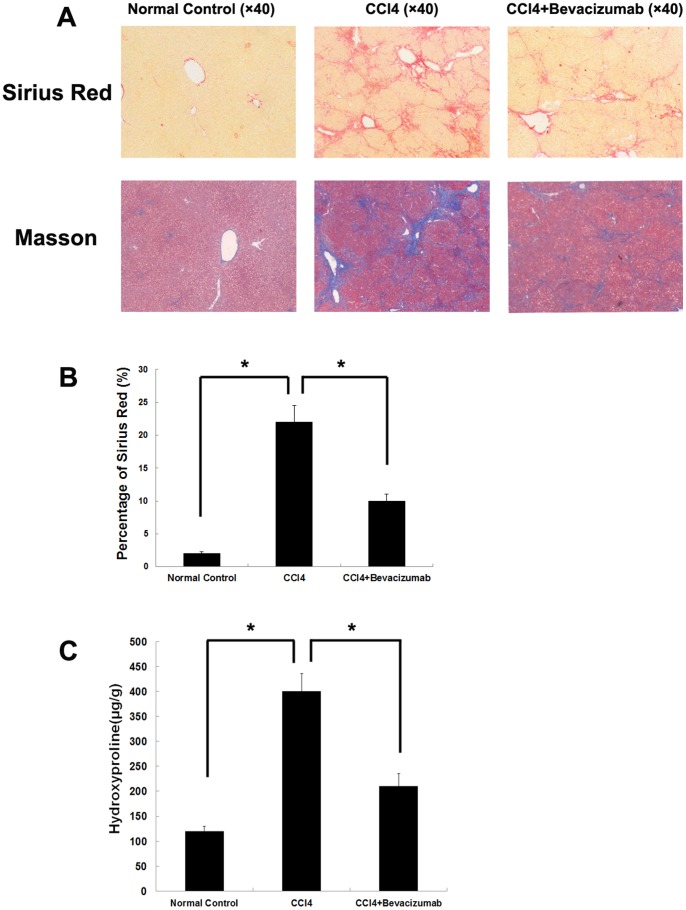
Bevacizumab attenuates hepatic fibrosis induced by CCl_4_ in rats. CCl_4_ was used to construct a hepatic fibrosis model to evaluate the therapeutic effects of bevacizumab(n = 8 for each group). (A) Sirius red and Masson's trichrome staining were used to determine the amount of ECM in the liver tissue of each groups. (B) Semiquantitative analysis of the ECM area was performed to evaluate the relative amount Sirius-red in fibrotic tissue using an image analysis system. (C) The amount of ECM was quantitated by quantitative estimation of hydroxyproline content. (*P<0.05).

We assessed the effects of bevacizumab in a rat model of CCl_4_-induced hepatic fibrosis. As shown in Figure 1A, bevacizumab administration had significantly reduced in fibrosis deposition as demonstrated by Sirius red staining and Masson's trichrome staining relative to the control group. This confirmed that bevacizumab attenuated hepatic fibrosis induced by CCl_4_ in rats. Semiquantitative analysis of the ECM area revealed that bevacizumab significantly reduced the area of ECM (Sirius red staining) after injection in the CCl_4_-treated fibrotic livers(Figure 1B). In CCl_4_-induced models, quantitative estimation of hydroxyproline content in the fibrotic groups indicated that the hydroxyproline content in bevacizumab-treated rats was 202.78±38.56 µg/g, which was lower than that in the positive control group (404.13±37.1 µg/g, *P*<0.05) (Figure 1C).

**Figure 2:**
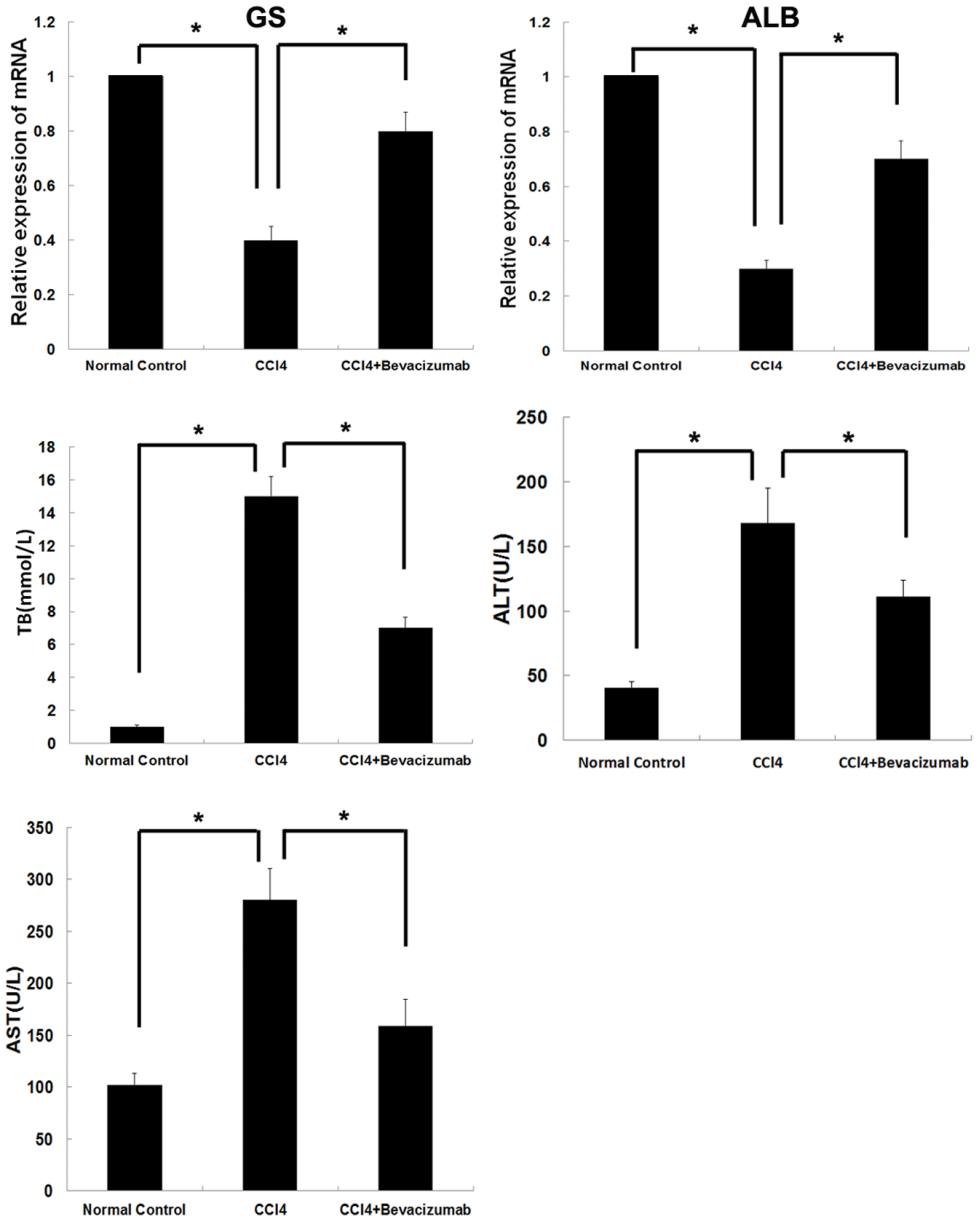
Effects of bevacizumab on liver function in fibrotic rats. The liver tissue and serum of the rats in each group was collected for assessment of liver function. GS, ALB, TB, AST and ALT were examined to assess hepatic function. Shown as the first two panels, GS and ALB levels were significantly lower of CCl_4_ group than that of normal control group. Bevacizumab administration significantly promoted synthesis of GS and ALB than that of CCl_4_ group. According to the left three panels, there were significantly differences of TB,ALT and AST levels among three groups. (*P<0.05).

As shown in Figure 2, bevacizumab ameliorated liver function in the fibrotic rats. Bevacizumab delivery significantly improved albumin (ALB) and glutamine synthetase (GS) levels in rats with hepatic fibrosis. Total bilirubin (TB), aspartate aminotransferase (AST) and alanine aminotransferase (ALT) concentration in bevacizumab-treated rats presented an obvious decrease relative to the control group.

### Bevacizumab downregulated profibrogenic genes expression *in vivo*

**Figure 3:**
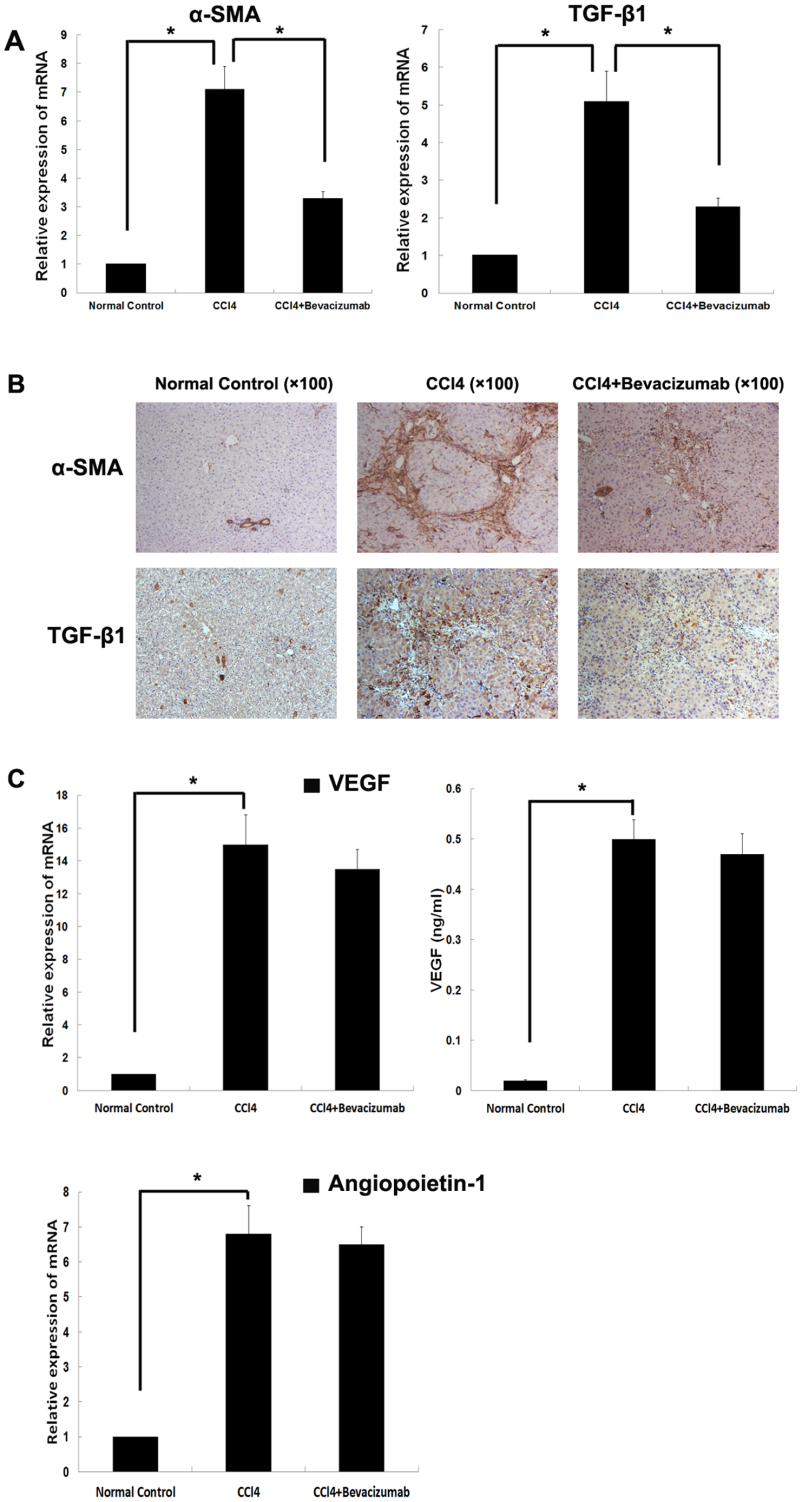
Effects of bevacizumab on profibrogenic gene expression *in vivo.* (A) Real-time PCR was used to assess the expression of α-SMA and TGF-β1, which have been reported to be important to the development of hepatic fibrosis. (B) Immunohistochemistry was used to examine α-SMA and TGF-β1 expression in liver tissues. (C) The expression of VEGF and angiopoietin-1 was examined by using real-time PCR and ELISA in the liver and serum. (*P<0.05).

Real-time PCR and immunohistochemical staining assays (IHC) were used to detect the expression of α-smooth muscle actin (α-SMA) and transforming growth factor-β1 (TGF-β1) in the liver tissues. These have been reported to be important for the development of hepatic fibrosis. The results demonstrated that expression of α-SMA and TGF-β1 was very low in normal liver tissue. However, a significant increase in gene expression was observed with the development of hepatic fibrosis induced by CCl_4_. Bevacizumab injection was found to remarkably down-regulate the expression of the genes related to liver fibrosis relative to the control groups (Figures 3A and B).

We also examined the expression of VEGF in liver tissue and serum. As shown in Figure 3C, the expression of VEGF was absent in normal liver tissue and serum, but up-regulation of VEGF was observed in hepatic fibrosis liver tissue and serum. Furthermore, we observed the VEGF level in liver and serum of hepatic fibrosis rats which have been administrated with bevacizumab. However, bevacizumab did not lead to a significant down-regulation of VEGF in the fibrosis liver. Beside that, we detected the expression of angiopoietin-1, another important angiogenesis associated factor, in liver. We could observe a obvious up-regulation of angiopoietin-1 in hepatic fibrosis group compared with control group and bevacizumab did not lead to a down-regulation of angiopoietin-1 in fibrosis liver. These results suggest that hepatocytes might produce VEGF during the formation of hepatic fibrosis.

### CCl_4_ lead to up-regulation of VEGF in hepatocytes

**Figure 4:**
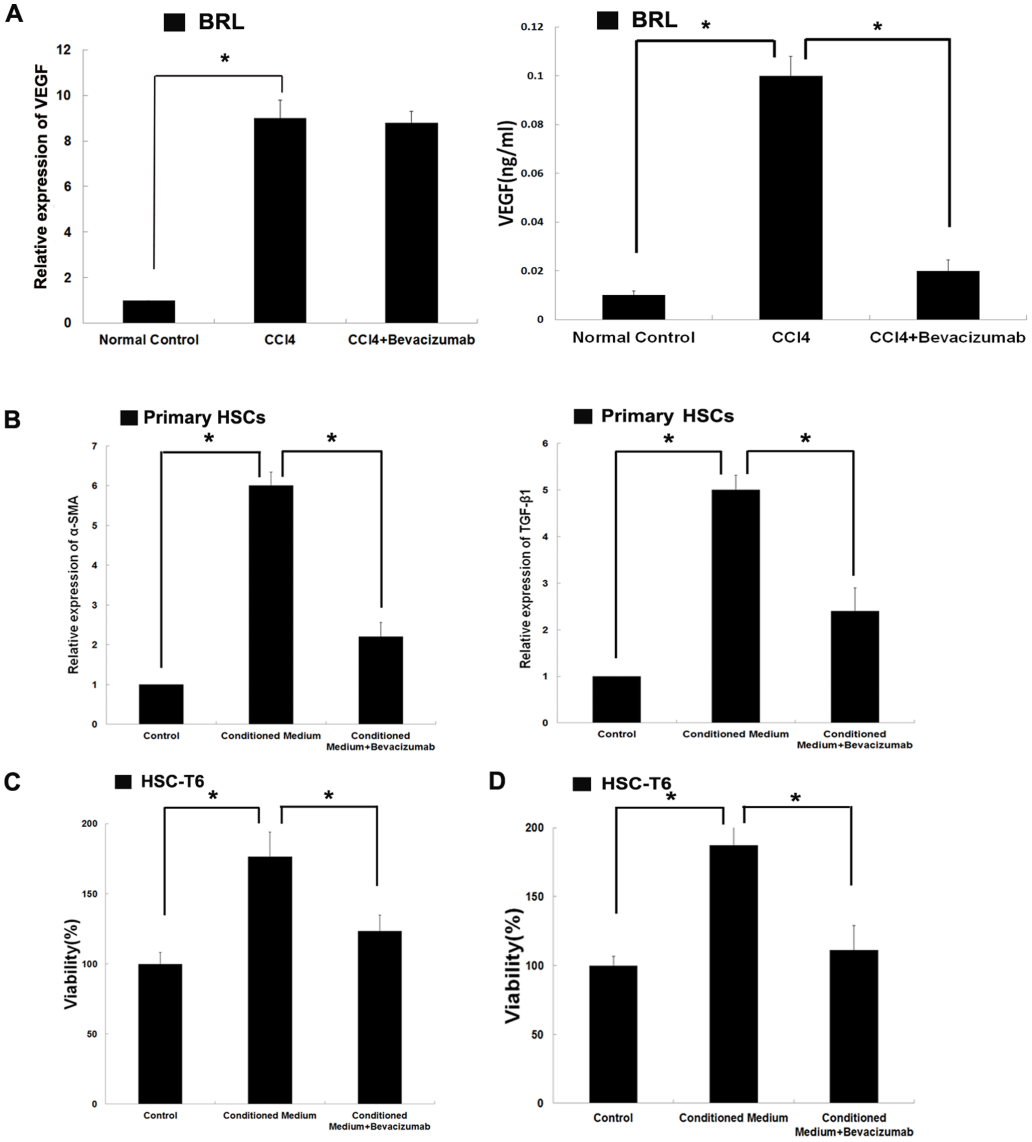
Effects of CCl_4_ on VEGF expression in hepatocytes and effects of conditioned medium collected from hepatocytes on the activation and proliferation of hepatic stellate cells. (A)BRL cells were exposed to CCl_4_ for 12 hours and then the culture medium was replaced with fresh DMEM. After another 24 hours of culture, the conditioned medium was collected. Real-time PCR and ELISA assays were used to assess VEGF expression in hepatocytes after exposure to CCl_4_. (B) BRL cells were exposed to CCl_4_ for 12 hours and then the culture medium was replaced with fresh DMEM. After another 24 hours of culture, the conditioned medium was collected. The primary HSCs were plated in 6-well plates (1×10^6^ cells/well) and treated by conditioned medium with or without bevacizumab (100 µg/ml) for 72 hours. Then the cells were harvested and real-time PCR was performed to assess the expression of α-SMA and TGF-β1 in HSCs. (C) CCK-8 assay was used to assess the effects of conditioned medium with or without bevacizumab (100 µg/ml) on the proliferation of the HSC-T6 cell line. (D) MTT assay was employed to examine the effects of conditioned medium with or without bevacizumab (100 µg/ml) on the proliferation of the HSC-T6 cell line. (*P<0.05).

The expression of VEGF was higher in fibrotic hepatic tissue than in healthy tissue. Real-time PCR and ELISA were used to examine VEGF expression in hepatocytes after exposure to CCl_4_. As shown in Figure 4A, there was a significant up-regulation of VEGF relative to the control group.

### Effects of conditioned medium collected from hepatocytes on the activation and proliferation of hepatic stellate cells

We have demonstrated that the expression of VEGF increased significantly during the formation of hepatic fibrosis and that bevacizumab could effectively attenuate hepatic fibrosis. HSCs have been shown to play a central role in the development of hepatic fibrosis. HSCs are considered a key target in anti-fibrotic therapy because of their role in ECM accumulation. The conditioned medium was collected from hepatocytes which were treated with CCl_4_. We then detected the gene expression of fibrotic markers in HSCs treated with the conditioned medium. As shown in Figure 4B, the expression of α-SMA and TGF-β1 in HSCs treated with conditioned medium was significantly higher than in self-activated HSCs. The HSC-T6 cell line was used to examine the effects of conditioned medium on the proliferation of HSCs. As shown in Figure 4C and D, conditioned medium promoted the proliferation of HSCs. These results indicated that the hepatocytes in fibrotic livers might play an important role in the activation of HSCs.

In order to confirm the contribution of VEGF to the activation of HSCs, bevacizumab was added in the conditioned medium, which was collected from hepatocytes exposed to CCl_4_. We found that the up-regulation of α-SMA and TGF-β1 in conditioned medium-treated HSCs was cancelled by bevacizumab (Figure 4B). In addition, bevacizumab blocked the enhancement of HSC-T6 cells proliferation caused by conditioned medium (Figure 4C and D). These results suggested that bevacizumab might be useful in preventing the activation and proliferation of HSCs during the development of hepatic fibrosis.

## Discussion

The hepatic fibrosis caused by many etiologies is an essential pathological process in chronic liver diseases and leads to loss of liver function and disrupts the structure of liver tissue[1], [2]. Angiogenesis is the main process of new vessel formation, accompanies with liver fibrosis and cirrhosis[5], [6], [24], [26], [27]. It can lead to generation of the new vessels from pre-existing blood vessels[3], [4]. Recently several studies reveal that angiogenesis plays a key role in fibrogenic progression of chronic liver diseases and the inhibition of pathological angiogenesis could regress or reverse liver fibrosis in experimental and clinical studies[4], [5], [6], [24], [26], [27]. The development of hepatic fibrosis was always associated with hypoxia and angiogenesis in hepatocytes[28], [29].

VEGF is the central angiogenic factor during chronic liver injury. The present study demonstrates that expression of VEGF-A is up-regulated in liver fibrosis, and its expression is increased in activated HSCs [5], [6], [8], [9]. Rosmorduc et al showed that biliary cirrhosis is associated with hepatocellular hypoxia in experimental models[8]. The expression of VEGF can be acticated by some hypoxic factor, such as hypoxia-inducible factor-1α (HIF-1α)[30]. Diethylnitrosamine induced chemical cirrhosis in rat demonstrates progressive hepatic fibrosis accompanied by up-regulation of VEGF and VEGF receptor and angiogenesis[29]. VEGF is produced by hepatocytes and induces hepatocellular growth by autocrine action[31]. Beside that, VEGF generated by hepatocytes also stimulates the proliferation of endothelial cells(ECs) in a paracrine fashion[32].

Bevacizumab has been proved to be a useful angiogenesis inhibitor. Its antiangiogenic efficacy is attributable to binding and neutralization of all isoforms of VEGF-A [18]. In this study, we investigated the effect of bevacizumab on the formation of hepatic fibrosis. We demonstrated that bevacizumab could effectively attenuate the development of hepatic fibrosis and contribute to the protection of liver function. Bevacizumab was also found to downregulate the expression α-SMA and TGF-β1, which have been reported to be profibrogenic genes *in vivo*. Furthermore, we observed the VEGF level in liver and serum of hepatic fibrosis rats which have been administrated with bevacizumab. However, bevacizumab did not lead to a significant down-regulation of VEGF. This result implied that bevacizumab may work by neutralizing VEGF rather than directly inhibiting the expression of VEGF in the fibrosis liver. These observations indicated that bevacizumab might inhibit the development of pathological angiogenesis in fibrosis tissue and influence the progress of liver fibrosis.

The activation of resident HSCs has been shown to play a central role in the development of hepatic fibrosis. HSCs are key target in anti-fibrotic therapy because of their function in ECM accumulation. HSCs underwent activation and conversion to myofibroblast-like cells capable of producing collagens and aggravating the deposition of ECM. Inflammatory mediators cause HSCs to differentiate into myofibroblasts and play a role in angiogenesis by releasing proangiogenic mediators, VEGF, and angiopoietin-1 during the development of liver fibrosis [16], [17]. On one hand, HSCs can behave as proangiogenic cells able to react to conditions of hypoxia by up-regulating transcription and synthesis of VEGF and its receptors[15], [17], [33]. On the other hand, HSCs are target cells for the action of VEGF. Hypoxia-dependent up-regulation of VEGF produced by HSCs demonstrates a paracrine and/or autocrine manner[17].

We showed that the expression of VEGF increased significantly during the development of hepatic fibrosis. The conditioned medium collected from hepatocytes exposed to CCl_4_ was found to activate the expression of fibrotic markers in HSCs and promote the proliferation of HSCs. We also observed that the expression of VEGF increased significantly during the development of hepatic fibrosis and that CCl_4_ could cause hepatocytes to produce VEGF. Bevacizumab neutralized VEGF significantly, which led to the blockage of the effect of VEGF produced by hepatocytes.

In conclusion, the results of our study suggested that bevacizumab might alleviate liver fibrosis by neutralizing VEGF produced by hepatocytes and block their effects on the activation of HSCs. Bevacizumab might be suitable as a potential agent for hepatic fibrosis therapy.

## Materials and Methods

### Cells and animals

Bevacizumab was obtained from Roche Ltd. (Shanghai, China). A rat normal liver cell line-BRL and an activated HSCs cell line-HSC-T6 were obtained from the cell bank of the Chinese Academy of Sciences (Shanghai, China).

Male Sprague-Dawley rats (190±15 g) were housed under standard animal laboratory conditions in the specific-pathogen-free-grade animal room at the Experimental Animal Center of the Second Military Medical University. The rats had free access to standard rat chow and water. This study was approved by the Local Ethical Committee of the Second Military Medical University.

### Hepatic fibrosis model

The hepatic fibrosis model of SD rats was induced by subcutaneous injection of 40% CCl_4_ at a dose of 2.4 ml/kg twice per week for 8 weeks[34]. Twenty-four male Sprague-Dawley (SD) rats were randomly divided into 3 groups. The first group (n = 8) served as a normal control group. The rats in next two groups (n = 16) were hepatic fibrosis models. The second group served as positive control group, and the rats in the third group were given 200 µg/kg bevacizumab. Infusions were given via the tail vein twice a week for 4 weeks starting from the 5th week in group 3. The rats in groups 1 and 2 were infused equal volumes of saline. One week after the last infusion, the animals were sacrificed by CO_2_ exposure and liver tissues were harvested.

### Histological examination and immunohistological staining

All paraffin-embedded liver tissues were HE stained for histopathological examination. Sirius red staining and Masson's trichrome staining were used to assess collagen levels. The red-stained areas in the Sirius Red stained sections were assessed with an image analyzer (Image-Pro Plus, MediaCybernetics) for semiquantitative analysis. The percentage of the Sirius Red was used to demonstrate the differences in each groups. Immunohistochemical examinations were used to detect the expression of α-SMA (Sigma Chemicals, St. Louis, MO, U.S.), and TGF-β1 (Santa Cruz Biotechnology, Inc., Santa Cruz, CA, U.S.).

### Measurement of hepatic hydroxyproline content

Total hepatic hydoxyproline levels were determined in the hydrolysates of liver samples as described previously [35].

### Serum biochemical analysis

Serum alanine aminotransferase (ALT), aspartate aminotransferase (AST) and total bilirubin(TB)were assessed by the kits from Sigma-Aldrich.

### HSCs isolation and culture

Primary HSCs were freshly isolated as described previously [36]. Cells were cultured in Dulbecco's modified Eagle's medium (DMEM) supplemented with 10% fetal bovine serum (FBS) and incubated at 37°C, 5% CO_2_ in a humidified incubator.

### Conditioned medium

BRL cells were stimulated with CCl_4_(5 mmol/L) for 12 hours. Then the culture medium was replaced with fresh DMEM. After another 24 hours of culture, the conditioned medium was obtained by collection and 0.22 µm filtration of the supernatant medium from BRL cells.

### Enzyme linked immunosorbent assay

ELISA assays were performed using a commercial VEGF ELISA kit (R&D Systems, Minneapolis, MN, U.S.). Samples were diluted 10-fold in deionized water before the assay. Assays were performed in duplicate, and readings were compared using standard curves obtained with standard protein provided with the kit. Samples were collected in triplicate, and means and standard deviations were compared using the t-test.

### Cell counting kit-8 assay

The measurement of viable cell mass was assessed by Cell Counting Kit-8 (Dojindo, Japan). Cells (5×10^3^ cells/well) were seeded in 96-well plates for overnight, we changed the medium with conditioned medium and continued to culture these cells for 24 hours. When the treatment was completed, 10 µl solution of Cell Counting Kit-8 was added in each well. The plate was continuously incubated for 2 hours. Finally, the absorbance of sample taken from each well was measured by microplate reader (Synergy HT, Bio-Tek) at 450 nm.

### MTT colorimetric assay

In order to measure the effects of conditioned medium on the viability of HSC-T6 cells, the cells were seeded in 96-well plates at a density of 5×10^3^ cells/well for overnight. Then we changed the culture medium with conditioned medium and continued to culture these cells for 24 hours. Twenty microlitres of MTT (5 mg/ml) were added to each well and the plate was continuously incubated for 4 hours. The formazan crystals were dissolved in 200 µl of DMSO. Finally, the absorbance of sample taken from each well was measured by microplate reader (Synergy HT, Bio-Tek) at 490 nm.

### Real-time polymerase chain reaction

Total RNA was extracted from the cells using Trizol reagent (Invitrogen, Carlsbad, CA, U.S.). The cDNA was synthesized using MMLV reverse transcriptase (Promega, WI, U.S.) and 2 µg total RNA and oligo dT18-primers. Real-time PCR was performed in triplicate using a SYBR PrimeScript RT-PCR Kit (Takara, Dalian, China). Total RNA was normalized by endogenous β-actin mRNA. The level of mRNA expression is presented as fold change relative to an untreated control. The primer sequences used in realtime-PCR are shown in Table 1.

**Table 1:** Oligonucleotide sequences of primers used in real-time PCR.

Gene	Sequence (5′→3′)
α-SMA	F: 5′-CCGAGATCTCACCGACTACC-3′
	R: 5′-TCCAGAGCGACATAGCACAG-3′
TGF-β1	F: 5′-ACCGCAACAACGCAATCTATG -3′
	R: 5′- ATTCCGTCTCCTTGGTTCAGC-3′
VEGF	F: 5′- CAGGGTTTCGGGAACTAG-3′
	R: 5′- GTGTATGTGGGTGGGTGT -3′
Angiopoietin-1	F: 5′-TACAACACCGTGAGGATGGA-3′
	R: 5′-TATCAGCGTCCTTTGTGCTG -3′
GS	F: 5′-AAGAGGGCATAGCCCAGACT-3′
	R: 5′-TTGGAAGCTTCGTTGGTCTT-3′
ALB	F: 5′-TGCAGGCTTGCTGTGATAAG-3′
	R: 5′-AGTAATCGGGGTGCCTTCTT-3′
β-actin	F: 5′-ACCCACACTGTGCCCATCTATG-3′
	R: 5′-AGAGTACTTGCGCTCAGGAGGA-3′

### Statistical Analysis

Data sets were analyzed by analysis of variance (ANOVA) with a posteriori contrast by least significant difference for comparisons among multiple groups and by Student t-test for comparison between two groups using the Microsoft Excel Analysis Tool Pak (Microsoft, Redmond, WA). The data, collected from at least three separate experiments, was showed as mean ±SEM. *P<0.05* was considered to be statistically significant.
